# The Structure of the RLIP76 RhoGAP-Ral Binding Domain Dyad: Fixed Position of the Domains Leads to Dual Engagement of Small G Proteins at the Membrane

**DOI:** 10.1016/j.str.2013.09.007

**Published:** 2013-12-03

**Authors:** Karthik V. Rajasekar, Louise J. Campbell, Daniel Nietlispach, Darerca Owen, Helen R. Mott

**Affiliations:** 1 Department of Biochemistry, University of Cambridge, 80 Tennis Court Road, Cambridge CB2 1GA, UK

## Abstract

RLIP76 is an effector for Ral small GTPases, which in turn lie downstream of the master regulator Ras. Evidence is growing that Ral and RLIP76 play a role in tumorigenesis, invasion, and metastasis. RLIP76 contains both a RhoGAP domain and a Ral binding domain (GBD) and is, therefore, a node between Ras and Rho family signaling. The structure of the RhoGAP-GBD dyad reveals that the RLIP76 RhoGAP domain adopts a canonical RhoGAP domain structure and that the linker between the two RLIP76 domains is structured, fixing the orientation of the two domains and allowing RLIP76 to interact with Rho-family GTPases and Ral simultaneously. However, the juxtaposed domains do not influence each other functionally, suggesting that the RLIP76-Ral interaction controls cellular localization and that the fixed orientation of the two domains orientates the RhoGAP domain with respect to the membrane, allowing it to be perfectly poised to engage its target G proteins.

## Highlights

• The structure of the RLIP76 RhoGAP-Ral binding domain dyad has been solved
• The interdomain linker contacts both domains and fixes their orientation
• The RhoGAP domain is a poor GAP for Cdc42 and Rac1 in vitro
• Ral and Rho family proteins can interact simultaneously with RLIP76

## Graphical Abstract

**Figure:**
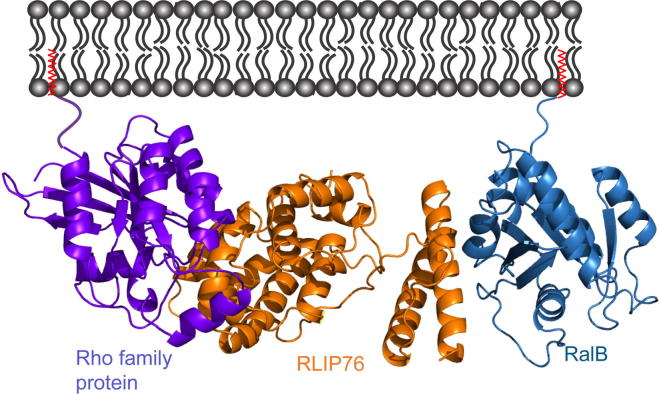

## Teaser

RLIP76 is an effector for Ral small GTPases that contains a RhoGAP domain adjacent to a Ral-binding domain. Rajasekar et al. solve a structure and reveal that the linker between the two domains is structured, fixing their orientation. This orientation allows them to engage their target G proteins.

## Introduction

The Ral interacting protein (RLIP76)/Ral binding protein (RalBP1) is a downstream effector of the Ral GTPases, which themselves lie downstream of the key regulator small G protein Ras. Activated Ras has the ability to initiate a cascade of signaling pathways due to its capacity to interact with several different groups of effector proteins, the best studied of which are the Rafs, PI3 kinases, and RalGEFs. Despite the supreme ability of Ras to transform cells, of all the Ras effector families, only the RalGEFs can transform immortalized primary human fibroblasts, and this activity requires Ral (Hamad et al., 2002). RalGEF and Ral are also known to be critical for an aggressive, metastatic phenotype in both 3T3 cells and bladder carcinoma cell lines (T24T) (Gildea et al., 2002; Ward et al., 2001). Hence, the RalGEF/Ral pathway is a potential target for the treatment of human cancers. The components of the RalGEF pathway downstream of Ral that mediate invasion and metastasis are starting to be elucidated. As RLIP76 is one of the effector proteins of the Ral GTPases, this makes it an important target to investigate, especially as there is evidence to suggest that RLIP76 has a role to play in cell motility (Coon et al., 2010) and that RalB and RLIP76 are responsible for the formation of invadopodia (Neel et al., 2012).

RLIP76 is a multidomain, multifunctional protein that was first identified for its ability to interact with activated Ral GTPases (Jullien-Flores et al., 1995; Park and Weinberg, 1995; Cantor et al., 1995). The Ral binding domain (GBD) of RLIP76 mediates the interaction with RalA/B, utilizing a coiled coil to contact the nucleotide-sensitive switch regions of the G protein (Fenwick et al., 2010). Immediately N terminal to the GBD is a region homologous to the GTPase-activating protein (GAP) domains for Rho GTPases, which has limited activity toward some members of the Rho family of small GTPases that are known to regulate actin cytoskeletal rearrangements and gene expression (Jullien-Flores et al., 1995; Park and Weinberg, 1995; Cantor et al., 1995). RLIP76 has also been linked to endocytosis and tyrosine kinase receptor signaling via its ability to bind to AP2, REPS1/REPS2 (POB1), and Epsin (Jullien-Flores et al., 2000; Yamaguchi et al., 1997; Ikeda et al., 1998; Coon et al., 2010). It can therefore be postulated that RLIP76 links Rho-family small G proteins to the endocytic machinery. RLIP76 has been shown to be associated with the active Cdk1 complex and serves as a platform for Cdk1 to phosphorylate Epsin and shut down endocytosis during mitosis (Rossé et al., 2003). Our knowledge of the roles of RLIP76 and RalA in mitosis has been extended more recently, as both proteins have been implicated in mitochondrial fission (Kashatus et al., 2011): RalA phosphorylation by AuroraA leads to RalA and RLIP76 localization to mitochondria and, once there, they regulate the phosphorylation of the Drp1 GTPase by Cdk1-CyclinB.

RLIP76 is usually located in the cytoplasm, but translocates to the membrane upon activation by Ral proteins (Lim et al., 2010). It contains two putative ATP binding sites (residues 65–80 and 415–448) (Awasthi et al., 2001), which allow membrane-associated RLIP76 to function as an ATP-dependent transporter protein that acts as an efflux pump for small molecules including anticancer drugs and endogenous metabolites (Vatsyayan et al., 2010). RLIP76 is overexpressed in metastatic bladder cancer (Smith et al., 2007), melanoma, lung carcinoma, and ovarian carcinoma (reviewed in Vatsyayan et al., 2010) and is necessary for metastasis of human pancreatic and bladder cancer cell lines in nude mice (Wu et al., 2010). RalB and RLIP76 together are required for the formation of invadopodia in pancreatic ductal adenocarcinoma (Neel et al., 2012). It has also been shown that overexpression of the RLIP76 GBD in MDCK cells represses the action of endogenous Ral proteins and that this repression leads to cell-cycle arrest and loss of anchorage-independent proliferation in human breast adenocarcinomas (Chien and White, 2003).

Whether RLIP76 functions as a modular protein with independent domains, so that each domain is free to interact with its own interacting partner(s), or whether the domains interact among themselves and regulate each other is currently unknown. As the RhoGAP domain interacts with Rho-family small G proteins and the GBD interacts with the Ral proteins, RLIP76 could act as an intersection point for these distinct signaling pathways, and juxtaposition of the GAP and GBD domains could be the strategy employed by RLIP76 to coordinate regulation of both pathways.

We have solved the structure of the RLIP76 GAP-GBD “didomain” with the aim of understanding any communication between the juxtaposed domains. The GBD in the dyad retains the same structure as the free GBD, and the RhoGAP domain adopts the canonical RhoGAP conformation, allowing the identification of the crucial catalytic residues. However, as RLIP76 has very poor GAP activity, we have also investigated the catalytic activity of the GAP domain and GAP-GBD didomain in vitro. Unexpectedly, the structure shows that the linker between the two domains is well defined and in a fixed conformation, sandwiching the two domains together in a locked configuration with respect to each other. Despite this, we find no evidence that either domain influences the functional activity of the other, and suggest rather that Ral binding to the RLIP76 GBD serves to localize RLIP76 to the correct cellular membrane and simultaneously fixes the orientation of the RLIP76 RhoGAP domain relative to that membrane, such that it is perfectly positioned to engage its Rho-family substrate, which will be tethered at the same location.

## Results

### Structure of the RLIP76 GAP-GBD Didomain

**Figure 1:**
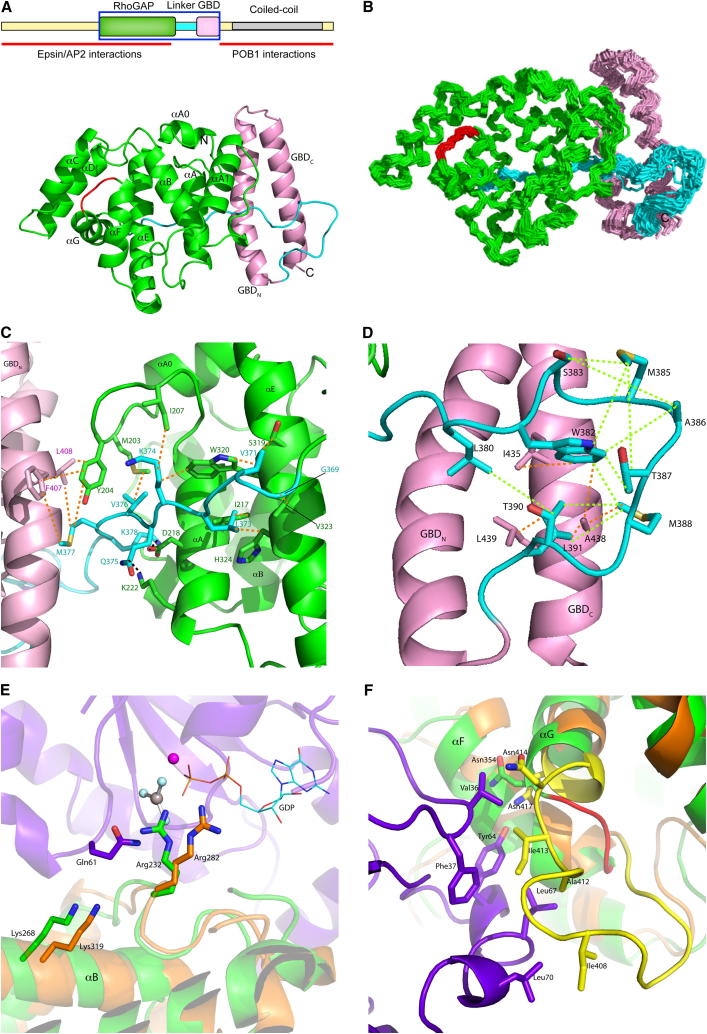
Structure of the RLIP76 GAP-GBD Didomain (A) The schematic domain structure of RLIP76 is shown above the closest structure to the mean. The limits of the construct whose structure is shown are outlined in blue. In this and all subsequent figures, the RhoGAP domain is colored green, the linker between the domains is cyan, and the GBD is pink. The loop between helices αF and αG is colored red. (B) The 35 lowest-energy structures calculated of the RLIP76 didomain. (C) Summary of the assigned distance restraints between the N-terminal half of the linker and the RhoGAP-GBD. Distance restraints were observed between the atoms connected by orange dashed lines. For clarity, only a single restraint for each residue pair is shown. Contacts between Asp218 and Lys222 in helix αA and the linker are shown as black dashed lines. (D) Summary of the assigned distance restraints between the hairpin of the linker and GBD and within the linker hairpin. Distance restraints were observed between the linker and the GBD for the atoms connected by orange dashed lines. The hairpin in the linker is defined by a number of distance restraints between atoms, shown as green dashed lines. For clarity, only a single restraint for each pair of residues is shown. (E) Comparison of the p50 RhoGAP-Cdc42 complex and the RLIP76 GAP domain. Cdc42 is purple, RLIP76 GAP is green, and p50 RhoGAP is orange. GDP is shown in a wire-frame representation, the Mg^2+^ ion is pink, and BeF_3_ is shown in a ball-and-stick representation. The arginine finger at the active site and the secondary lysine are oriented correctly in the RLIP76 GAP domain to aid catalysis. The positions of Arg232^RLIP76^ and Arg282^p50^ are the same, and the side chains are oriented in the same direction in both RLIP76 and the p50 RhoGAP-Cdc42 transition-state complex (PDB ID code 1GRN). Similarly, Lys268^RLIP76^ is oriented so that it can support the position of the arginine finger loop in a similar manner to Lys319^p50^. (F) The loop between helices αF and αG is truncated in the RLIP76 GAP domain. The RLIP76 GAP domain (in green) has a shorter loop (shown in red) between the two helices. In the complex formed between Cdc42·GMPPNP (purple) and p50 RhoGAP (orange), several contacts are made by the longer loop (shown in yellow). The only contact that remains is at the beginning of the αG helix, where Asn414 in p50 RhoGAP makes a hydrogen bond with Tyr64. The equivalent residue in RLIP76 is Asn354, but this is not sufficient for a high-affinity interaction. The residues involved in interactions between Cdc42 and p50 RhoGAP are shown in a stick representation.

The resonances of the GAP-GBD didomain (residues 184–446) were assigned as reported elsewhere (Rajasekar et al., 2012). The final ensemble of structures was calculated from a total of 8,551 nuclear Overhauser effect (NOE) restraints, which were translated by ARIA into 4,748 unambiguous and 2,272 ambiguous unique restraints. Except for part of the loop region that connects the two domains, the structure is well defined (Figures 1A and 1B), with a root-mean-square deviation (rmsd) of 0.76 Å (Table 1).

**Figure 2:**
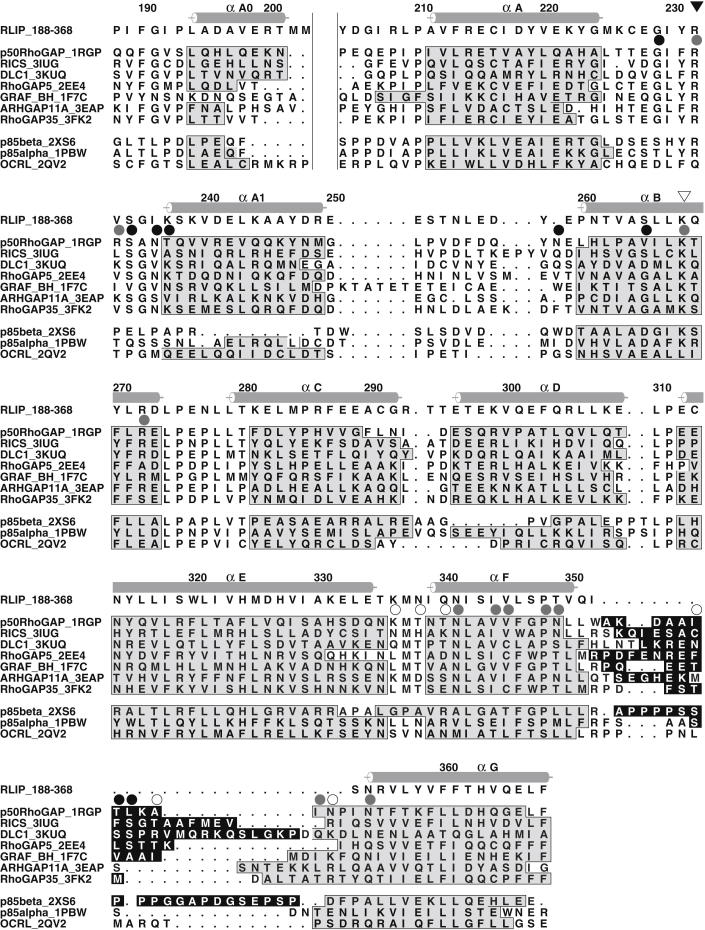
Sequence Alignment of RLIP76 with RhoGAP Domains Whose Structures Have Been Solved The positions of the RLIP76 α helices are denoted as gray cylinders above the alignment and are labeled according to the names of the equivalent helices in the p50 RhoGAP protein (Barrett et al., 1997). The helical regions in all the proteins are shaded gray and boxed. Residues involved in contacts with Cdc42 are marked by circles above the p50 RhoGAP sequence colored as follows: white circles: in contact in the Cdc42·GMPPNP complex (PDB ID code 1AM4); black circles: in contact in the Cdc42·GDP·AlF_3_ complex (PDB ID code 1GRN); gray circles: in contact in both complexes. The Arg finger and secondary Lys are marked by black and white triangles, respectively. Residues in the loop between helices αF and αG are shown in white font on a black background if their backbone B factors in the X-ray structures are more than 20 Å^2^ higher than the average or whose coordinates are missing. For the NMR structure of RhoGAP5, residues with a backbone rmsd of more than 2 Å are highlighted in the same manner. The two vertical lines between helices A0 and A mark the position of a large insertion in OCRL, which has been omitted for clarity. The alignment was generated using T-Coffee (Di Tommaso et al., 2011) and Top3d (Winn et al., 2011) and the figure was produced using ALSCRIPT (Barton, 1993). The PDB ID codes for the structures are shown after the name of each protein.

The GAP domain (residues 184–368) is formed by nine α helices arranged in a typical RhoGAP fold (Barrett et al., 1997; Rittinger et al., 1997), usually named αA0, αA, αA1, and αB-αG (Figure 2). The core of the domain is formed by helices A, B, E, and F, which form a four-helical bundle. Helices C and D sandwich helix G onto the face of helices E and F, and helix A1 packs against helices A and B. The conserved arginine “finger,” Arg232, is present in the loop between helices A and A1. The secondary lysine, Lys268, which stabilizes the position of the finger loop, is within helix B. Comparison of the RLIP76 GAP domain structure with the canonical RhoGAP shows that the orientation of the α helices is conserved, with an rmsd of ∼1.5 Å when the helical regions are overlaid.

The GBD (residues 393–446) in the didomain is a coiled coil, which is similar to the structure that we previously observed both in isolation and in complex with RalB (Fenwick et al., 2010). The backbone rmsd between the two GBD α helices in this structure and in the RalB complex (Protein Data Bank [PDB] ID code 2KWI) is ∼1.0 Å. Thus, the overall architecture of the G protein binding domain is preserved in the longer construct. This is in agreement with our observations that the GAP-GBD didomain and the GBD single domain bind to RalB with the same affinity of ∼200 nM (Fenwick et al., 2010 and data not shown).

The orientation of the two single domains that make up the GAP-GBD didomain is well defined in the family of structures due to a number of distance restraints observed between each domain and the linker that connects them. The final α helix of the canonical RhoGAP domain finishes at Phe368, and the next nine residues (Gly369–Met377) contact the surface of the GAP domain comprising helices αE and αA and the linker between αA0 and αA (Figure 1C). Several of the residues involved in these interactions are hydrophobic and not conserved in the other RhoGAP proteins, for example, Met203, Ile207, and Val323, which are absent, Glu257, and Ala372, respectively, in p50 RhoGAP (Figure 2). Met377 also interacts with two exposed hydrophobic residues in the N-terminal α helix of the GBD, Phe407 and Leu408. Trp382–Met388 forms a turn in the linker that reverses the chain and brings it back toward the GBD (Figure 1D). The residues in the center of this hairpin are flexible and the backbone amides of residues 383, 384, and 385 could not be assigned, implying that they are undergoing dynamic changes on a millisecond timescale. The ends of the hairpin are buttressed against the GBD by interactions between Trp382 and Met388 in the linker and Ile435 and Ala438 in the C-terminal helix of the GBD. The hairpin itself is defined by multiple distance restraints, for example, from Trp382 and Ser383 to Met385, Ala386, Thr387, and Met388. The remainder of the linker is pinned against the GBD by interactions between Leu391 and Ala438 and Leu439 in the GBD.

There are few distance restraints between the two domains because they do not form a large buried surface area at their interface, and indeed only come together where Met377 in the linker acts as a hasp between the GAP and the N-terminal α helix of the GBD. Met377 contacts Tyr204, which is sandwiched between Phe407 and Leu408, and distance restraints between Met377 and Phe407 reinforce the contacts (Figure 1C). Long-range data from paramagnetic relaxation enhancement (PRE) experiments support the location of the contacts between the domains: attachment of a spin label to Cys411 in the GBD leads to complete bleaching of residues in helix αA0 and in the αA0-αA loop.

### The RLIP76 GAP Domain Displays Weak GAP Activity toward Cdc42 and Rac1

**Figure 3:**
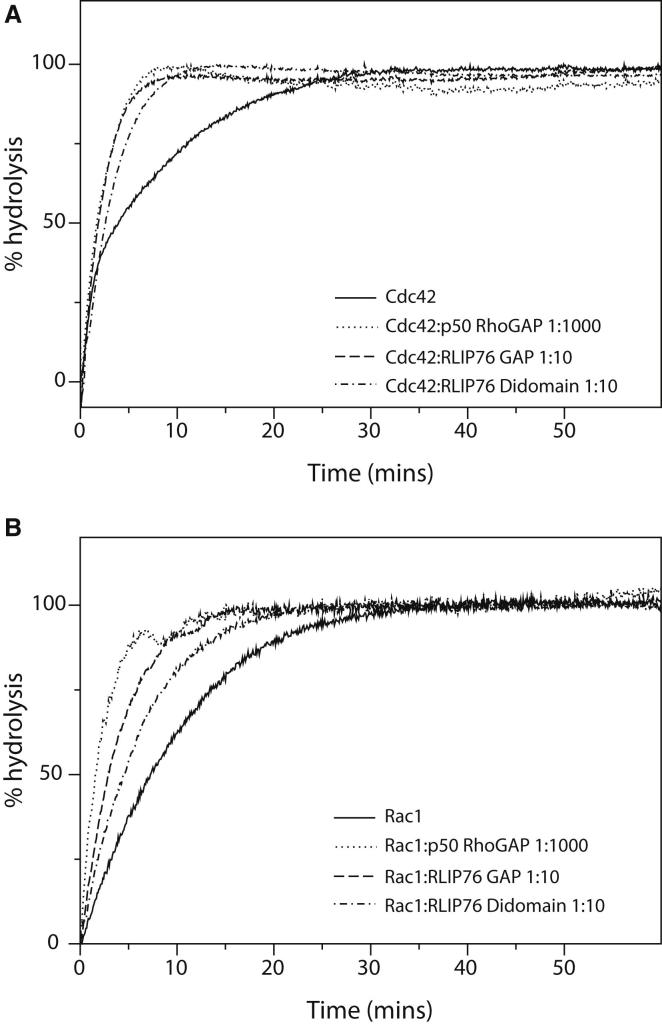
Real-Time GAP Assays with RLIP76 and p50 RhoGAP The intrinsic GTP hydrolysis and the hydrolysis stimulated by p50 RhoGAP, RLIP76 GAP domain, and the RLIP76 didomain are shown. (A) GTP hydrolysis by Cdc42. (B) GTP hydrolysis by Rac1.

The GAP activity of the RLIP76 GAP domain and the RLIP76 didomain were determined in vitro using a real-time GAP assay. Wild-type Rac1 and Cdc42 were preloaded with GTP, and the hydrolysis of the nucleotide bound to the G protein was measured by assaying for the phosphate released (Figure 3; Table 2). The intrinsic rates of GTP hydrolysis measured for Rac1 and Cdc42 were 0.097 and 0.148 min^−1^, respectively, similar to values previously obtained by real-time experiments (Zhang et al., 1997, 1998). Addition of the bona fide GAP domain from p50 RhoGAP at low levels (25 nM) stimulated the GTPase activity of both Cdc42 and Rac1, as expected. Addition of the RLIP76 RhoGAP domain at the same concentrations had no discernible effect on the GTP hydrolysis rate of either Rac1 or Cdc42 (data not shown), but when RLIP76 was added at higher concentrations, such as 2.5 μM, stimulation of the GTPase activity could be observed (Figure 3). Overall, the ability of the RLIP76 GAP domain to stimulate the GTPase activity of Cdc42 and Rac1 is >100-fold less efficient than the stimulatory effect of p50 RhoGAP.

To test the possibility that the juxtaposed GBD was necessary for efficient GAP activity, we also performed GAP assays using the GAP-GBD didomain at 2.5 μM and compared its activity toward Rac1 and Cdc42. There was no increase in the activity with the didomain when compared to the GAP domain alone. Indeed, there is a small but reproducible decrease in the stimulation of GTP hydrolysis by the didomain (Table 2). Addition of stoichiometric quantities of GMPPNP-loaded RalB had no effect on the GAP activity of the didomain (data not shown), in line with previous work on RLIP76 (Jullien-Flores et al., 1995; Park and Weinberg, 1995; Cantor et al., 1995).

Thus, although the Rho family GAP domain in RLIP76 can be identified by sequence homology, we have shown that its stimulation of hydrolysis by Rac1 and Cdc42 is significantly weaker in vitro than that of their bona fide GAP, p50 RhoGAP. The sequence alignments (Figure 2) and structural alignment around the active site (Figure 1E) show that a conserved pair of basic residues, the “arginine finger” and the Lys that supports the position of the arginine finger loop, is present in RLIP76. These residues are crucial for Ras- and RhoGAP-mediated hydrolysis reactions and are in the correct position for stimulation of catalysis to be possible in RLIP76. The presence of the linker and GBD appear to have a slight inhibitory effect on the GAP domain, which was not relieved by the presence of the GBD binding partner RalB.

### Why Does RLIP76 Have Low Catalytic Activity?

**Figure 4:**
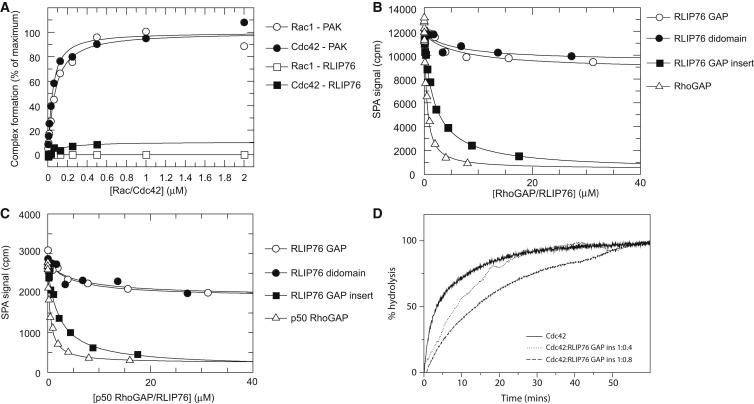
Biochemical Analysis of the RLIP76 GAP Domain and the GAP Insert Mutant (A) Scintillation proximity assays. The indicated concentrations of [^3^H]GTP·Rac1 and Cdc42 were incubated with 70 nM His-RLIP76 didomain or GST-PAK1 in SPAs. The SPA signal was corrected by subtraction of a blank from which the fusion protein was omitted. The effect of the [G protein] on this corrected SPA counts/min signal was fitted to a binding isotherm to give an apparent K_d_ value and the signal at saturating concentrations of G protein. The data are expressed as a percentage of this maximum signal. (B and C) Competition SPAs. Displacement of (B) [^3^H]GTP·Cdc42 or (C) [^3^H]GTP·Rac1 from GST-p50 RhoGAP by RLIP76 constructs or by untagged p50 RhoGAP. Increasing concentrations of RLIP76 or p50 RhoGAP proteins were titrated into fixed concentrations of [^3^H]GTP·Cdc42 or [^3^H]GTP·Rac1 and GST-p50 RhoGAP. Fits of the data to a partial competition model for RLIP76 GAP or RLIP76 didomain binding to Cdc42 (B) or Rac1 (C) are shown. The K_d_ value for Cdc42 or Rac1 binding to GST-p50 RhoGAP was fixed to the values obtained from direct SPAs (80 and 86 nM, respectively). For the RLIP76 GAP insert or p50 RhoGAP, full competition was used to produce the fits shown. For p50 RhoGAP, the K_d_ values obtained by competition SPA were 247 ± 30 nM and 150 ± 12 nM for Cdc42 and Rac1, respectively. (D) Real-time GAP assays with the RLIP76 GAP insert. The rates of GTP hydrolysis by Cdc42 alone and in the presence of different concentrations of the RLIP76 GAP insert. The integrity of the insert protein was established by gel filtration, western blotting, and circular dichroism (see Figure S1).

As the basic catalytic machinery appears to be present in the RLIP76 GAP domain, we examined the RLIP76 RhoGAP domain structure for features that might contribute to its low activity. Comparison of the sequences of RhoGAP domains whose structures are known (Figure 2) shows that between helices αF and αG there is a loop that, in the free RhoGAP proteins, is highly dynamic. In the free p50 RhoGAP structure, part of the loop is absent in the structure due to its mobility, whereas in the other free RhoGAP domain structures there are either missing coordinates or very high temperature factors for residues in the loop. In RLIP76, the loop is much shorter, as is the N terminus of helix αG (Figure 2), and our NMR dynamics analysis shows that the three residues that remain are rigid (data not shown). When p50 RhoGAP forms a complex with Cdc42 and stimulates the GTPase activity, this loop binds to residues in the switch 2 region of Cdc42 and becomes rigid (Figure 1F). The essential contacts between this αF-αG loop of p50 RhoGAP and Cdc42 have been defined by our previous mutagenesis analysis of the Cdc42-RhoGAP interaction (Owen et al., 2000, 2008). The residues at the center of the loop interface, Phe37^Cdc42^, Tyr64^Cdc42^, and Leu67^Cdc42^, when mutated, reduced the binding 6-fold (F37A), 9-fold (L67A), and >20-fold (Y64A). Mutations of Val36^Cdc42^ and Leu70^Cdc42^, which are at the periphery of the loop interface, do not affect the binding affinity, indicating that although they make contacts with the GAP domain, they are not thermodynamically essential. These data highlight the importance of the interactions between the switch regions of Cdc42 and the αF-αG loop of p50 RhoGAP for successful formation of the complex. None of these contacts can be formed by RLIP76. We therefore reasoned that the shortened loop in RLIP76 might lead to an inability of the GAP domain to engage Cdc42 and Rac1, whereas p50 RhoGAP binds to Cdc42 and Rac1 with affinities of 24 and 18 nM, respectively (Owen et al., 2008). We measured the binding affinity of RLIP76 GAP for Rac1 and Cdc42 using scintillation proximity assays (SPAs), where His-tagged RLIP76 didomain or GAP domain was bound to protein A-coated SPA beads via an anti-His antibody. An SPA signal is obtained when binding of His-RLIP76 to Cdc42 (or Rac1)·[^3^H]GTP occurs. Experiments were performed using the GTPase-deficient mutants Q61L Cdc42/Rac1 so that stable [^3^H]GTP complexes could be obtained. Affinities were determined for the interactions between the His-RLIP76 didomain or the His-GAP domain and both Cdc42 and Rac1. There was no discernable binding between either Cdc42 or Rac1 with the RLIP76 didomain or the GAP domain (Figure 4A) even though both G proteins bound to the positive control, PAK, with affinities of 32 nM (Cdc42) and 57 nM (Rac1), which are similar to values we have measured previously (Owen et al., 2000, 2003).

We endeavored to determine whether reinstating a longer loop between helices αF and αG in RLIP76 could restore Cdc42 binding and thus improve RLIP76 GAP activity. Residues Gln351–Ser353 (Q-I-S) of the RLIP76 GAP domain were replaced by the equivalent sequence Leu401–Ile416 in p50 RhoGAP (L-W-A-K-D-A-A-I-T-L-K-A-I-N-P-I). The refolded protein was assessed by circular dichroism and shown to be helical (Figure S1 available online). The binding affinity of the RLIP76 GAP insert for Cdc42 and Rac1 was measured by competition SPA, which can measure lower affinities than direct SPA. Proteins were assayed by binding to Rac1 or Cdc42·[^3^H]GTP that had been prebound to GST-tagged, immobilized p50 RhoGAP. We first measured the binding affinity of RLIP76 GAP and RLIP76 didomain using competition SPA to quantify the weak affinity of native RLIP76 for Cdc42/Rac1. Interestingly, at saturating concentrations of RLIP76 RhoGAP domain or RhoGAP-GBD didomain, the signal did not return to the experimentally determined zero (i.e., the signal obtained in the absence of GST effector), which would be expected for a full competitor. The differences observable between the partial competitors RLIP76 GAP and RLIP76 didomain and a pure competitor, such as free p50 RhoGAP itself, are apparent (Figures 4B and 4C). This observation prompted us to fit the data using an equation describing partial inhibition, in which the competitor modulates the affinity of the monitored reaction but does not abolish it completely. In this scenario, even at saturating levels of RLIP76, there is residual GST-RhoGAP-Cdc42/Rac1 complex, which gives rise to the SPA signal observed. The K_d_ values for RLIP76 GAP and RLIP76 didomain binding to Cdc42 and Rac1 derived from fitting to a partial competition model are shown in Table 3. The partial competition by RLIP76 indicates that the binding surface on Cdc42 and Rac1 for the RLIP76 GAP domain does not fully overlap the binding surface for the p50 RhoGAP domain. This supports our hypothesis, implying that the αF-αG loop of p50 RhoGAP mediates some residual binding to Cdc42 and Rac1 even in the presence of high concentrations of RLIP76 GAP, allowing a ternary complex to form. In contrast, when the competition assay was performed using the RLIP76 GAP insert, the signal returned to the experimentally determined zero at saturating concentrations, indicating pure competition (Figures 4B and 4C). This implies that introduction of the longer αF-αG loop results in a larger binding interface more closely resembling that between Cdc42 and p50 RhoGAP. For Cdc42, although the affinity is still weaker for the RLIP76 GAP insert than for p50 RhoGAP, it is significantly higher than for the native RLIP76 GAP protein (Table 3). Interestingly, native RLIP76 GAP and didomain bind to Rac1 more tightly than Cdc42 (around 1 μM K_d_), and the affinity between Rac1 and the RLIP76 GAP insert was essentially the same (Table 3). As we do not have a Rac1-p50 RhoGAP structure, it is difficult to speculate on differences that must exist between the Cdc42-p50 RhoGAP and Rac1-p50 RhoGAP complexes. However, RLIP76 is still a poor GAP for Rac1, despite having some affinity for this G protein. It is notable that the bona fide RhoGAP, p50 RhoGAP, binds to both Cdc42 and Rac1 with nanomolar affinity.

The RLIP76 GAP insert was then tested in real-time GAP assays at low concentrations (25 nM), but it had no effect on the Cdc42 GAP activity. When higher concentrations of the RLIP76 GAP insert domain were used, close to stoichiometric levels compared to the Cdc42 concentration, the RLIP76 GAP insert actually inhibited the GTPase when compared to its intrinsic rate of hydrolysis (Figure 4D). The intrinsic observed rate was 0.16 min^−1^; when 0.4 molar equivalents of the RLIP76 GAP insert were added the rate decreased to 0.084 min^−1^, and when 0.8 molar equivalents were added the rate decreased to 0.048 min^−1^.

Taken together, these data demonstrate that engineering the αF-αG loop from p50 RhoGAP into the RLIP76 GAP domain is sufficient to impart a measurable increase in binding affinity of RLIP76 GAP for Cdc42. Nevertheless, even this engineered form of RLIP76 GAP, which binds more tightly to Cdc42, cannot behave as an efficient GAP. Furthermore, the stabilization of the Cdc42 switch regions by the αF-αG loop actually inhibits the GTPase activity when it is present in RLIP76 GAP, presumably because it binds Cdc42·GTP to form an unproductive complex.

### Modeling Protein-Protein Interactions at the Rho-Ras Signaling Node

**Figure 5:**
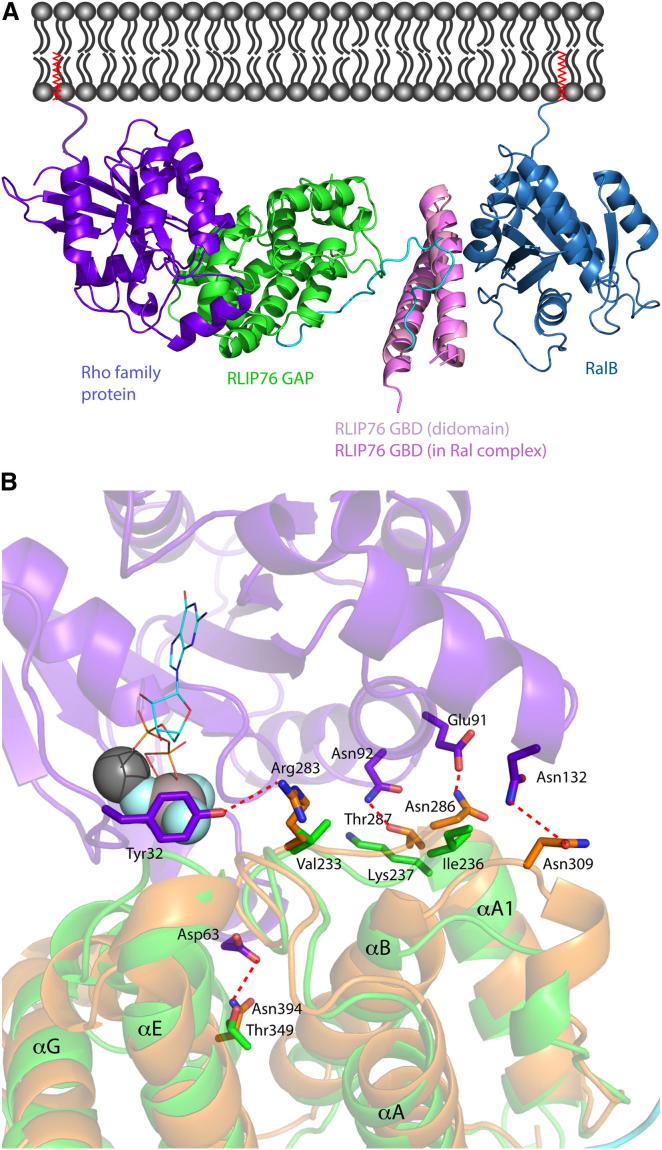
RLIP76 GAP-GBD Interactions with Ral and Rho Small G Proteins (A) The RLIP76 GAP-GBD didomain can simultaneously interact with Ral and Rho family proteins. The model of the Cdc42·GMPPNP-didomain complex was superimposed over the RLIP76 GBD onto the RLIP76-RalB complex structure. Cdc42 is purple-blue, the RLIP76 GAP domain is green, the linker between the GAP and GBD is cyan, the GBD is in two shades of pink, and RalB is sky blue. The binding of Cdc42 and RalB together is possible because the binding sites are on the exterior faces of the two domains that make up the didomain. The orientation of the two G proteins is such that their C-terminal helices are pointing toward one side of the trimer and their C-terminal isoprenyl groups (red) will be able to simultaneously engage the lipid bilayer (gray). (B) Interactions in the Cdc42·GDP·AlF_3_-p50 RhoGAP transition-state complex that would not be conserved in the equivalent complex with RLIP76. The secondary structures of Cdc42 (purple), RLIP76 GAP (green), and p50 RhoGAP (orange) are semitransparent for clarity. The GDP is shown in a wire-frame representation, Mg^2+^ is a dark gray sphere, and AlF_3_ is a light gray sphere surrounded by three pale blue spheres. Side chains of Cdc42 and p50 RhoGAP involved in interactions are shown in a stick representation and colored in the same scheme as the secondary structures. The equivalent residues in RLIP76 GAP are also shown as sticks.

Solving the structure of the RLIP76 GAP-GBD didomain allowed us to construct a model of the tripartite complex that would form between RLIP76, Cdc42, and RalB. We used the p50 RhoGAP-Cdc42 structure (PDB ID code 1AM4) to dock Cdc42 to the RLIP76 GAP domain. The GBD in this model was then superimposed onto the GBD in our structure of the RalB-GBD complex (PDB ID code 2KWI). The resulting complex model (Figure 5A) shows that the binding of a Rho-family protein and Ral proteins is not mutually exclusive but rather that simultaneous binding of two G proteins at the membrane is facilitated by the fixed orientation of the two domains. This is borne out by our binding studies that show that the GBD and didomain have the same affinity for RalB, namely that the presence of the GAP domain does not affect Ral binding. Even though the GBD is only a small coiled coil, its interactions with the GAP domain and the linker between the domains mainly involve the N-terminal helix of the GBD, whereas the interaction with RalB is predominantly with the C-terminal helix. The only region of the GAP-GBD didomain that would potentially clash with RalB is the tip of the hairpin within the linker region. The residues in this hairpin are already known to be flexible from the NMR dynamics analysis, and they would therefore be able to move to accommodate the binding of RalB. The Rho protein binds to a concave surface of the GAP domain on the opposite face to the GBD, and it would appear that the presence of GBD should not affect Rho protein binding at all.

The two G proteins are oriented in the model such that their C termini are on the same side of the heterotrimer, which has important potential functional consequences in vivo. The Cdc42 and RalB complex structures were solved with truncated proteins, which are therefore not posttranslationally modified, as they would be in vivo. In both full-length G proteins, the α helix at the C terminus is followed by a flexible tail with a lipid moiety attached, which is responsible for membrane attachment. Cdc42 and RalB in the heterotrimer model are oriented such that they would be able to be simultaneously membrane bound via their C termini (Figure 5A). Our model therefore demonstrates that the tripartite complex would satisfy the restraints imposed on these proteins in vivo and allow a possible scaffold function for RLIP76.

## Discussion

The structure that we present here describes the only two recognized domains of RLIP76. Due to their respective binding partners, the RhoGAP domain-Ral binding domain dyad represents the physical point of confluence of Ras and Rho signaling at RLIP76.

Our structure demonstrates that the RhoGAP domain of RLIP76 contains the conserved elements that would usually confer catalytic GTPase activation on the target G proteins. Despite this, RLIP76, even with respect to those small G proteins toward which it has some activity, is only a very poor GAP.

To try to understand the molecular basis of the catalytic activity of RLIP76, we examined other regions of p50 RhoGAP that contact Cdc42 in the transition-state complex (Nassar et al., 1998) and are not conserved in RLIP76 (Figure 2). These include Arg283^p50^, which forms a weak hydrogen bond with Tyr32^Cdc42^ and is replaced by Val233 in RLIP76 (Figure 5B). Mutation of Tyr32^Cdc42^ can prevent GAP-stimulated hydrolysis of GTP without reducing binding of Cdc42 and p50 RhoGAP (Fidyk and Cerione, 2002), suggesting that it is key to the formation of the transition state. Asn286 and Thr287 of p50 RhoGAP are replaced by Ile236 and Lys237 in RLIP76. Asn286^p50^ forms a hydrogen bond with Glu91^Cdc42^ in the transition-state complex, whereas Thr287^p50^ makes a hydrogen bond with Asn92^Cdc42^. The replacement of Asn286^p50^ by the hydrophobic Ile residue in RLIP76 is likely to change the interaction with this region significantly. The equivalent residues in Graf RhoGAP (Val224 and Asn225; Figure 2) have been mutated and appear to be involved in selectivity between different GTPases (Jelen et al., 2009). It is likely therefore that RLIP76 forms different contacts in the transition state of the reaction and that this underpins its altered activity to Cdc42. Thus, the structure adds to the general body of information regarding the action of GAP domains, highlighting the point that the orientation of side chains important to the transition state are as important as the canonical catalytic residues.

The structure of the RhoGAP domain of RLIP76 reveals the truncation of a flexible loop that is involved in substrate binding in p50 RhoGAP. The absence of this loop goes some way to explaining the low-level activity of RLIP76 toward Cdc42, as addition of the loop restores some binding ability for this substrate. However, engineering this loop into the RLIP76 RhoGAP domain does not elevate GAP activity toward Cdc42 or Rac. It is possible that the presence of the loop from p50 RhoGAP actually hinders formation of the correct transition state, because at high concentrations the RLIP76 mutant with the loop inserted slightly inhibited the Cdc42 GTPase activity.

This raises the question of the true activity of the RLIP76 GAP domain in vivo. As all of our work has utilized the isolated RhoGAP domain or RhoGAP-GBD didomain, it is possible that, in full-length RLIP76, regions of the protein outside the GAP domain could contribute residues that impart high-affinity binding to Cdc42. A second possibility is that the true substrate for RLIP76 has yet to be identified. Sequence analysis shows that the Rho protein binding sites on p50 RhoGAP and RLIP76 are significantly different. As there are 22 members of the Rho family of GTPases, it would seem likely that the molecular details of engagement of substrates by RhoGAP domains would be different, permitting novel modes of substrate engagement by different RhoGAP domains. Only a systematic analysis of all of the Rho family members will help us to understand the function of this domain and identify true substrates. These two possibilities, however, are not mutually exclusive, and we are currently investigating both. It is also possible that RLIP76 could have evolved to function as a weak GAP, so that its function is married to its localization to the membrane by the GBD interacting with Ral proteins. Thus, it would only be active as a GAP when the substrate concentration is high or, for example, when the substrate G protein is posttranslationally modified and membrane attached. This is consistent with in vivo data that show that Cdc42 is a target for *Xenopus laevis* RLIP76 (Boissel et al., 2007) and data in cell lines that show that RNAi knockdown of RLIP76 leads to increased levels of Cdc42·GTP and Rac1·GTP (Lim et al., 2010).

A final consideration is that the RLIP76 GAP domain could be a binding module for a different family of GTPases, although as the main catalytic residues are present and correctly aligned, this seems unlikely. There is, however, precedence for such a function, such as the RasGAP domain in IQGAP, which displays no catalytic activity and has subverted the catalytic domain to utilize as a binding motif for Rho-family G proteins (Owen et al., 2008), and OCRL, which contains a nonfunctional RhoGAP domain that interacts with endocytic adaptors (Pirruccello et al., 2011). Neither IQGAP nor OCRL contains an Arg finger, but the RhoGAP domains from the regulatory subunits of the class 1A PI3 kinases p85α and p85β include both the Arg finger and a secondary Lys and are still considered to be nonfunctional as GAPs.

The original driver for this work was to determine any influence these two small G protein binding domains had toward each other. The structure of the didomain shows that the two domains are in a fixed orientation with respect to one another. The linker between them packs between the two domains, fastening them together, and thus it is easily conceivable that communication between the two is possible. We therefore first examined the GAP activity of the RLIP76 GAP domain both alone and within the context of the didomain. The didomain displays a small (∼30%) but reproducible decrease in activity in comparison to the GAP domain alone. Although it does not seem likely that such a small decrease would be functionally significant in vivo, it does show that the RLIP76 GAP domain has the capacity for slightly higher activity in the absence of the GBD. The GBD makes no contacts with the catalytic residues in the GAP domain, so we assume that this influence is a long-range, allosteric effect that prevents the subtle rearrangements important for formation of the transition state of the reaction. We also examined the effect of the GAP domain on the ability of the GBD to bind its partner, RalB. The binding affinities of the GBD and the didomain for RalB are identical, so the ability of the GBD to bind Ral proteins is unaffected by the presence of the GAP domain.

As we expected any influence of each domain on the other to be regulatable, we investigated whether the activity was altered as a consequence of the presence of binding partners. The GAP activity of the didomain toward Cdc42 and Rac1 remains unaltered in the presence of RalB; that is, binding of RalB to the GBD does not alleviate the small negative influence of the GBD on the GAP activity. We cannot exclude the possibility, however, that the presence of Ral proteins might regulate the activity of the GAP domain toward other, as yet unidentified, Rho-family proteins.

Thus, it would seem that although the two domains of RLIP76 are both juxtaposed and tethered in a fixed conformation, neither seems to have a large influence over the known functions of the other. We therefore conclude that the function of the GBD is to bind to one of the two Ral isoforms, which then acts to localize RLIP76 to a particular cellular compartment. At its particular location it then encounters the relevant substrate for the GAP domain. Our model of the tripartite complex reveals that the orientations of the domains would facilitate membrane localization by Ral with synchronous binding of Rho-family substrates by RLIP76.

## Experimental Procedures

### Protein Expression and Purification

Human RLIP76 GAP (residues 184–380) and GAP-GBD domains (residues 184–446) were expressed and purified as described previously (Rajasekar et al., 2012).

The F-G loop from p50 RhoGAP was inserted into the pET16b RLIP GAP domain construct using site-directed mutagenesis. Residues ^351^QIS^353^ of RLIP76 were replaced with ^401^LWAKDAAITLKAINPI^417^ from p50 RhoGAP. Mutagenesis was performed using a QuikChange Lightning mutagenesis kit (Agilent), following the manufacturer’s instructions. The RLIP76 GAP domain with the inserted F-G loop from p50 RhoGAP was expressed in *Escherichia coli* BL21 (DE3) in inclusion bodies, which were washed in 2% Triton and 2 M NaCl and solubilized by resuspension in 6 M GuHCl (pH 7.5). The protein was refolded by rapid, 50× dilution at 4°C into 1.5 M 1-(3-sulfopropyl)-pyridinium betaine (Raschig-GmbH), stirred at 4°C overnight, concentrated, and further purified on a 16/60 S75 column.

Mutations of cysteine residues in the GAP-GBD didomain were carried out using the QuikChange Lightning Multi Site-Directed mutagenesis kit (Agilent) following the manufacturer’s instructions. Cys216 and Cys227 were mutated to alanine; Cys291 and Cys313 were mutated to serine.

All other expression constructs have been described elsewhere (Owen et al., 2000).

### NMR and Structure Generation

All NMR experiments were collected and analyzed as described (Rajasekar et al., 2012). Backbone torsion angles were estimated from CA, CO, CB, N, and HA chemical shifts using the program TALOS+ (Shen et al., 2009). CCPN ANALYSIS (Vranken et al., 2005) was used to generate distance restraints from NOESY spectra. Structures were calculated using ARIA 1.2 (Linge et al., 2003) interfaced to CNS (Brünger et al., 1998), where the ambiguity of the restraints was decreased during eight iterations.

Residual dipolar couplings (RDCs) were measured on a Bruker DRX600 by partially aligning the protein in 13 mg/ml Pf1 phage (Asla Biotech). RDCs were measured by comparing peak positions in TROSY and decoupled ^15^N-HSQC experiments or by using the ARTSY method (Fitzkee and Bax, 2010). Initial values for the alignment tensors were estimated using a histogram of the observed dipolar couplings (Clore et al., 1998). The values were refined with a grid search using a modified version of ARIA 1.2 (Houben et al., 2004; H.R.M., unpublished data).

PRE restraints were measured using a mutant protein that had a single cysteine remaining at position 411. MTSSL (1-oxyl-2,2,5,5-tetramethylpyrroline-3-methyl) methanethiosulfonate; Enzo LifeSciences) was attached to Cys411 by incubating the ^15^N-labeled protein with 20 mM MTSSL at 4°C overnight in 20 mM Tris-HCl (pH 7.9) and 100 mM NaCl. MTSSL loading was confirmed by mass spectrometry. ^15^N-TROSY HSQC spectra were recorded on unlabeled and MTSSL-labeled protein. Cross-peak intensities were measured, and the ratios were used to generate distance restraints (Battiste and Wagner, 2000) between 16 and 23 Å with errors of ±10 Å. Cross-peaks that had disappeared in the MTSSL-labeled spectra were restrained to 16 Å with no lower bound and the upper bound set to 26 Å.

### In Vitro GAP Assays

Rac1 and Cdc42 were GTP loaded by incubation in a 10× molar excess of GTP, 60 mM ammonium sulfate, 20 mM EDTA, 20 mM Tris-HCl (pH 7.4), and 50 mM NaCl for 1 min at 37°C. Then the mixture was briefly incubated on ice and MgCl_2_ was added to 100 mM. The reaction mixture was passed through a G25 spin column to remove excess GTP and used immediately in the assays. The release of phosphate from Rac1 or Cdc42 was monitored at 360 nm using the EnzChek phosphate assay kit (Invitrogen) to quantify inorganic phosphate released in the reaction. The GAP activities of the RLIP76 didomain, RLIP76 GAP domain, and the GAP domain from p50-RhoGAP toward wild-type Rac1 (25 μM) and wild-type Cdc42 (25 μM) were monitored simultaneously on a microplate spectrophotometer (Spectra Max Plus; Molecular Devices). All experiments were performed at 25°C in 50 mM Tris-HCl (pH 7.5), 10 mM NaCl, and 1 mM MgCl_2_.

### Scintillation Proximity Assays

#### Direct Binding SPAs

Affinities of Rac1 and Cdc42 for His-RLIP76 GAP or His-RLIP76 didomain were measured using SPAs, in which the fusion protein was attached to a fluoromicrosphere via an anti-His antibody (Sigma) in the presence of Q61L Rac1/Cdc42·[^3^H]GTP. Binding of the G protein to the His-RLIP76 constructs brings the labeled nucleotide close enough to the scintillant to obtain a signal. Apparent K_d_s for Q61L Rac1/Cdc42·[^3^H]GTP were measured as described previously (Thompson et al., 1998) by varying the concentration of Rac1/Cdc42·[^3^H]GTP at a constant concentration of 70 nM His-RLIP76. Using this method, the upper and lower limits of the K_d_ that can accurately be measured are 1,000 and 1 nM, respectively. For each affinity determination, data points were obtained for at least ten different Rac1/Cdc42 concentrations. Binding curves were fitted using the appropriate binding isotherms to obtain K_d_ values and their standard errors (Thompson et al., 1998). The integrity of the Rac1/Cdc42·[^3^H]GTP proteins was checked using GST-PAK as a control binding partner as described previously (Owen et al., 2000).

#### Competition SPAs

For competition assays, His-RLIP76 GAP, His-RLIP76 GAP-GBD didomain, His-RLIP76 GAP insert, or free RhoGAP was titrated into a mixture of 30 nM [^3^H]GTP⋅Rac1 or [^3^H]GTP⋅Cdc42 and 30 nM GST-p50 RhoGAP immobilized on fluoromicrospheres via an anti-GST antibody (Invitrogen). The added RLIP76 or free p50 RhoGAP proteins compete with the GST-p50 RhoGAP-[^3^H]GTP⋅Rac1/Cdc42 interaction, abolishing the scintillation signal. The highest concentrations of competitor used were RhoGAP, 16 μM; His-RLIP76 GAP, 31.25 μM; His-RLIP76 didomain, 27.3 μM; and His-RLIP76 GAP insert, 17.5 μM. In each case, a blank was performed in the absence of GST-p50 RhoGAP GBD. For affinity determination, data points were obtained for at least ten different competitor concentrations. The K_d_ value and its standard errors were obtained by fitting the dose-response curve to binding isotherms that describe competition between two proteins binding to one site on another protein and account for mutual depletion of the interacting components. The K_d_ values for the GST-p50 RhoGAP-Rac1/Cdc42 interactions were obtained from direct-binding SPAs (Fenwick et al., 2009). The equations used were adapted for SPA from the previously published derivations (Wang, 1995) and have been fully described elsewhere (Elliot-Smith et al., 2007).

## Author Contributions

D.O. and H.R.M. conceived the project. L.J.C. made constructs and performed SPAs. K.V.R. made NMR samples and performed GAP assays. D.N. ran the NMR experiments. K.V.R. and H.R.M. analyzed NMR data and solved the structure. D.O. and H.R.M. characterized the GAP insert mutant. K.V.R., D.O., and H.R.M. wrote the manuscript.

## Figures and Tables

**Table 1:** Structural Statistics for the RLIP76 GAP-GBD Didomain

Experimental Restraints Used in Structure Calculation	
Unambiguous NOEs	4,748
Ambiguous NOEs	2,272
Dihedral angle restraints (ϕ + ψ)	472
Hydrogen bonds	118
PRE distance restraints	130
RDCs (phage)	141
GAP to linker NOEs	73
GAP to GBD NOEs	3
Linker to GBD NOEs	23
Intralinker NOEs (nonsequential)	33

Structural Statistics		
Coordinate precision (Å)	<SA>^a^	<SA>_c_^b^
RMSD of backbone atoms		
GAP-GBD didomain 190–444 (Å)	0.76	0.50
GAP domain 190–380 (Å)	0.56	0.40
GBD 395–444 (Å)	0.43	0.32
Rmsd of heavy atoms		
GAP-GBD didomain 190–444 (Å)	1.20	0.98
GAP 190–380 (Å)	1.02	0.89
GBD 395–444 (Å)	1.01	0.98
RMS deviations		
From experimental restraints		
All NOE distances (Å)	0.024 ± 0.0029	0.028
PRE distances (Å)	0.009 ± 0.0053	0.013
GAP to linker NOEs (Å)	0.022 ± 0.0056	0.021
GAP to GBD NOEs (Å)	0.051 ± 0.029	0.000
Linker to GBD NOEs (Å)	0.029 ± 0.011	0.041
Intralinker NOEs (Å)	0.031 ± 0.0099	0.036
Dihedral angles (^o^)	1.06 ± 011	0.969
RDC Q factor	0.242 ± 0.013^c^	0.242^c^
From idealized geometry		
Bonds (Å)	0.0039 ± 5.3 × 10^−5^	0.0038
Angles (^o^)	0.578 ± 0.011	0.558
Impropers (^o^)	1.53 ± 0.059	1.44
Ramachandran analysis^d^		
Most favored regions (%)	86	86.3
Allowed regions (%)	11.5	12.0
Generously allowed regions (%)	1.9	0.8
Disallowed regions (%)	0.6	0.8

a <SA> is the average rms deviation for the ensemble ± the standard deviation.

b <SA>_c_ is the value for the structure that is closest to the mean.

c Calculated using PALES (Zweckstetter, 2008): Q={∑i=1N[dinorm(exp)−dinorm(calc)]2/N}12/Dr.m.s., where *D*^*r.m.s*^. was calculated from the experimental couplings.

d PROCHECK (Laskowski et al., 1993).

**Table 2:** Real-Time GAP Assays Recorded on Cdc42 and Rac1

	Observed Rate (min^−1^)	Fold Stimulation (over Intrinsic)
Rac1 (intrinsic)	0.097 ± 0.0003	–
Rac1 + p50 RhoGAP^a^	0.404 ± 0.004	4.16
Rac1 + RLIP76 GAP^b^	0.238 ± 0.0007	2.45
Rac1 + RLIP76 didomain^b^	0.160 ± 0.0004	1.65
Cdc42 (intrinsic)	0.148 ± 0.001	–
Cdc42 + p50 RhoGAP^a^	0.490 ± 0.005	3.3
Cdc42 + RLIP76 GAP^a^	0.425 ± 0.003	2.87
Cdc42 + RLIP76 didomain^b^	0.304 ± 0.002	2.05

a p50 RhoGAP (25 nM) was added.

b RLIP76 GAP (2.5 μM) and didomain were added.

**Table 3:** Affinities of RLIP76 Constructs for Cdc42 and Rac1 Measured by Competition SPA

	Cdc42 (μM)	Rac1 (μM)
RLIP76 GAP	6.19 ± 3.35	1.68 ± 0.82
RLIP76 GAP-GBD didomain	5.59 ± 3.58	2.34 ± 1.32
RLIP76 GAP insert	1.27 ± 0.12	1.44 ± 0.25

## Accession Numbers

The coordinates and structural restraints for the RLIP76 RhoGAP-Ral binding domain dyad have been deposited in the PDB under ID code 2MBG. The assignments are available at BioMagResBank under accession number 17608.

## Supplemental Information

Document S1. Supplemental Experimental Procedures and Figure S1
