# Supplementary material for: The Structure of the RLIP76 RhoGAP-Ral Binding Domain Dyad: Fixed Position of the Domains Leads to Dual Engagement of Small G Proteins at the Membrane
Source: Structure. 2013 Dec 3;21(12):2131–42. doi: 10.1016/j.str.2013.09.007 (PMC3852207; doi:10.1016/j.str.2013.09.007)
Supplement: Document S1. Supplemental Experimental Procedures and Figure S1 [file mmc1.pdf]

Structure, Volume *21*

Supplemental Information

The Structure of the RLIP76 RhoGAP-Ral Binding Domain Dyad: Fixed Position of the Domains Leads to Dual Engagement of Small G Proteins at the Membrane

Karthik V. Rajasekar, Louise J. Campbell, Daniel Nietlispach, Darerca Owen, and Helen R. Mott

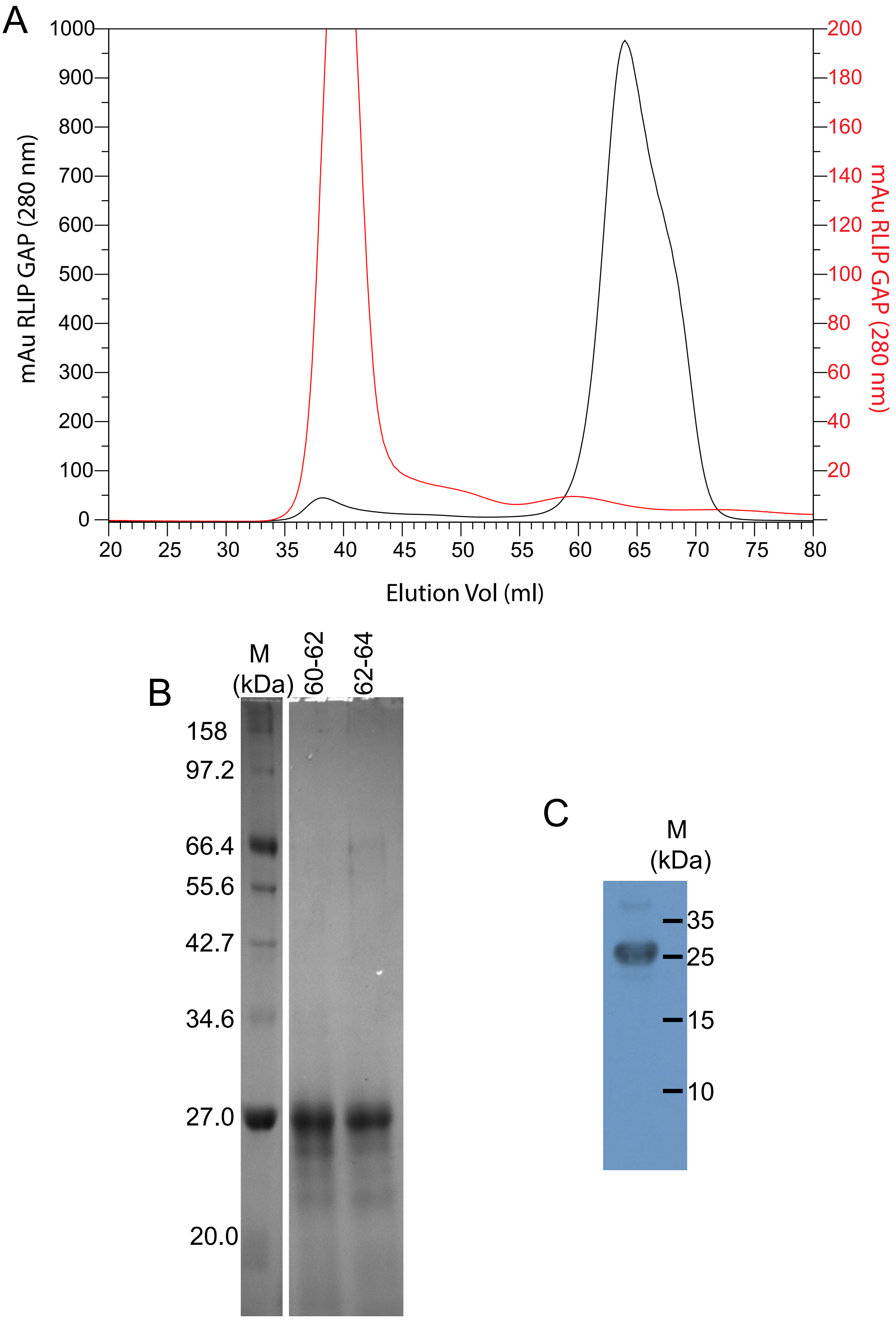

A
mAu RLIP GAP (280 nm)
mAu RLIP GAP (280 nm)
1000
900
800
700
600
500
400
300
200
100
0
200
180
160
140
120
100
80
60
40
20
20 25 30 35 40 45 50 55 60 65 70 75 80
Elution Vol (ml)
B
M (kDa)
60-62
62-64
158
97.2
66.4
55.6
42.7
34.6
27.0
20.0
C
M (kDa)
35
25
15
10

D

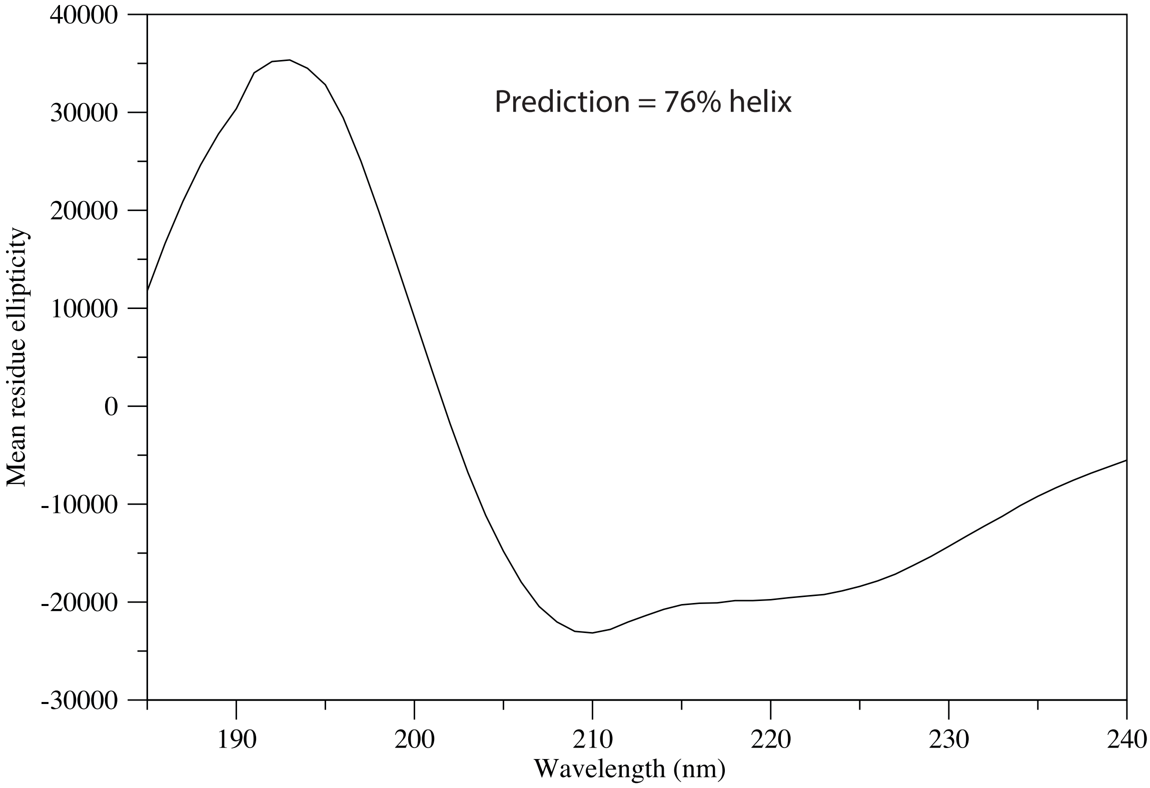
Prediction = 76% helix
Mean residue ellipticity
40000
30000
20000
10000
0
-10000
-20000
-30000
190
200
210
220
230
240
Wavelength (nm)

E

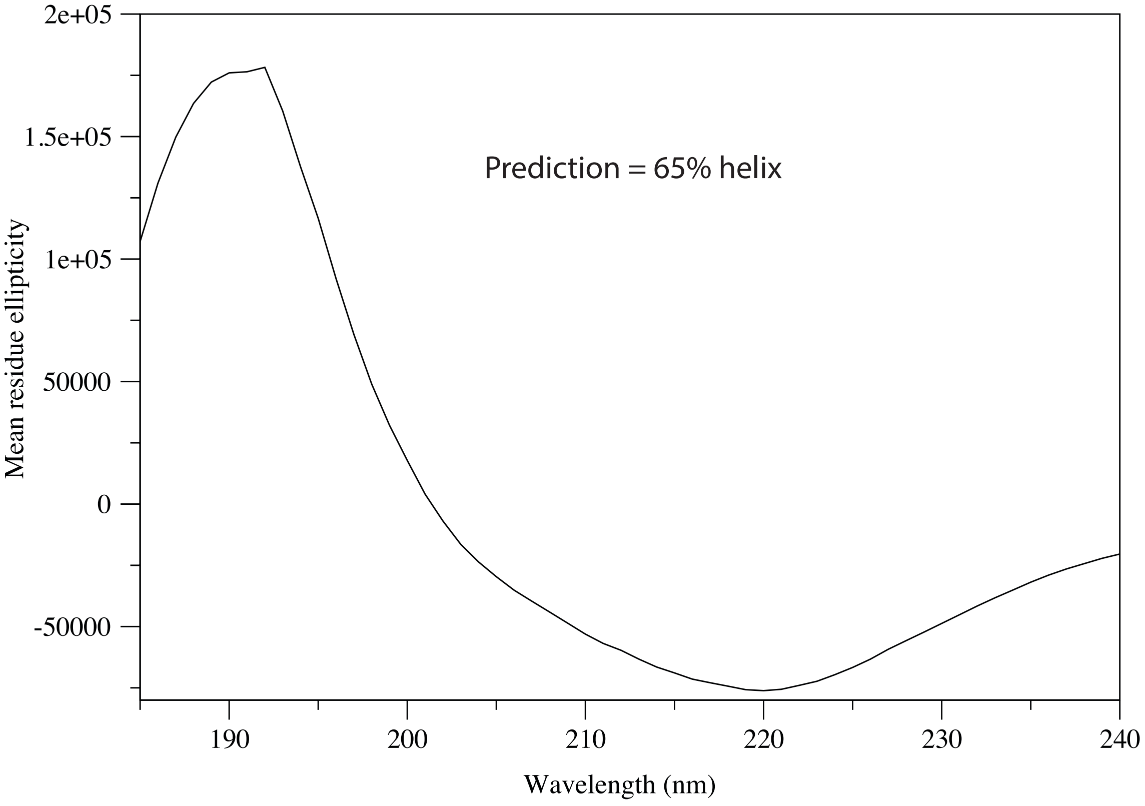
Prediction = 65% helix
Mean residue ellipticity
2e+05
1.5e+05
1e+05
50000
0
-50000
190
200
210
220
230
240
Wavelength (nm)

Supplementary Methods.
CD experiments were recorded in 50 mM sodium phosphate pH 7.8, 100 mM sodium fluoride. Spectra were recorded on an AVIV 410 in a 0.1 cm path length quartz cuvette. Three wavelength scans were recorded for each sample between 260 and 185 nm. Secondary structure prediction was performed using DICHROWEB (Whitmore & Wallace, 2004; Whitmore & Wallace, 2008), using CDSSTR (Compton & Johnson, 1986; Sreerama & Woody, 2000) and reference set 3.

Supplementary Figure S1
Purification of the RLIP GAP insert protein from inclusion bodies and CD analysis

A. Gel filtration traces of RLIP76 GAP domain (black) and refolded RLIP76 GAP insert (red). The RLIP76 GAP insert is shown scaled up 5-fold (y-axis scale on the right) compared to the RLIP76 GAP domain (y-axis scale on the left).
B. The individual 2 ml fractions were concentrated and ran on SDS-PAGE, followed by Coomassie staining. The two fractions that eluted between 60 and 64 ml contained a discrete band at 27 kDa and were pooled.
C. A Western blot probed with anti-His (Santa Cruz SC803) shows that the 27 kDa band includes a His-tag.
D. CD spectrum of RLIP76 GAP domain and the secondary structure prediction from the CD. The helicity predicted from our structure is 58%.
E. CD spectrum of GAP insert mutant protein and the secondary structure prediction from the CD. The helicity predicted from our structure is 55% if it is assumed that the insert loop is unstructured.

References.

Compton LA, Johnson WC (1986) Analysis of Protein Circular-Dichroism Spectra for Secondary Structure Using a Simple Matrix Multiplication. *Anal Biochem* **155:** 155-167

Sreerama N, Woody RW (2000) Analysis of protein CD spectra: Comparison of CONTIN, SELCON3, and CDSSTR methods in CDPro software. *Biophys J* **78:** 334A-334A

Whitmore L, Wallace BA (2004) DICHROWEB, an online server for protein secondary structure analyses from circular dichroism spectroscopic data. *Nucl Acid Res* **32:** W668-W673

Whitmore L, Wallace BA (2008) Protein secondary structure analyses from circular dichroism spectroscopy: Methods and reference databases. *Biopolymers* **89:** 392-400
